# The Week's Work

**Published:** 1910-08-20

**Authors:** 


### The Week's Work.

### THE LATE MISS FLORENCE NIGHTINGALE.

The event of the week is the death of the "Lady of the Lamp," whose sudden but not entirely unexpected death occurred on Saturday last. While Miss Nightingale's nursing work is known to all those who know her name, her association with the institutional world and her wide influence upon hospital life in this country are not perhaps so widely known as they deserve to be. Yet her hospital and institutional work was no less pioneering, no less beneficial than her nursing work at Scutari. She utilised the magnificent tribute of respect and admiration which the nation offered her to found and endow the Training School for Nurses at St. Thomas's Hospital—a school in which she always showed the keenest interest. She chose St. Thomas's as her base because of her admiration for, and appreciation of, the efforts of the then matron, Mrs. Wardroper, whose connection with the school ensured its success, although it is significant of the thoroughness of Miss Nightingale's methods that she insisted upon the matron—whose quarters, it is alleged, were in the monkey house at the old Surrey Gardens music-hall—undertaking to learn the duties of nursing from the first. Mrs. Wardroper became the first trained hospital nurse who graduated under the "Lady of the Lamp," and the fame of the Nightingale school was such that at one time of its history it supplied nurses and matrons to nearly every important hospital in the Empire. Now we have nursing and training schools on all sides, and a generation has arisen that knows not of its indebtedness to the magnificent energy, courage, and resourcefulness of Florence Nightingale. To-day, when the nursing and institutional world is mourning her death, it is fitting to pause for a moment and reflect upon the work that she initiated and the triumph that she achieved. Her permanent monuments are the nursing schools and the nursing system in this country. Like Wren's, they are round about you when you look, but infinitely greater than his, unbounded by walls, or limited by twelve pillars and a cross. In life she gently put aside all honours and worldly tributes of praise, and endured the enforced seclusion of her last years with a patient cheerfulness. She carries with her to the grave the respect and love, not only of the profession whose chief she was, but of a whole nation.

### A WORKHOUSE SCANDAL.

General hospitals have often experienced the censure of Boards of Guardians, and have had to content themselves with justifying their actions as best they could, well knowing that in the majority of cases the sympathy of the public is largely with the Guardians. The very nature of the case sometimes prevents the hospital secretary or superintendent from taking the Press fully into his confidence, and expatiating upon the reasons which prompted the authorities to send this or that patient to the workhouse. It is easy enough for the Guardians, when they hear that a patient was sent away from the hospital when he or she was in a precarious condition, to express their indignation at the inhumanity of the hospital officials, for it is only rarely when they have among them a lady or gentleman who is thoroughly acquainted with hospital routine, and who knows from bitter experience the distress which an out-patient officer or a taking-in official feels when he has to refuse a bad case owing to the fact that there is no possible room for it. We wish the superintendents would take the public more fully into their confidence, and actually show the young men of the Press, who come investigating around when a scandal is on the wind, to what shifts they are sometimes driven to take in a critical case. But in general Boards of Guardians seem to refuse to credit that a hospital is really humane, and only too ready to impute blame where only inability existed. Now and then, however, the Guardians have a scandal of their own, and it is possible that these unpleasant experiences, which are sometimes pressed home to them by a stern Local Government Board, may serve to make them more sympathetic to the harassed hospital worker, who is nearly always utterly guiltless of the criminality which is imputed to him. Recently, for example, the Southwark Guardians were severely reproved by Mr. Burns's department for having neglected to remove a patient from the workhouse to the infirmary when her condition made such removal imperatively necessary. This particular Board, we believe, has been especially severe upon the faults, the sins of omission and commission, of hospital workers in its district, and it is to be hoped that the letter from the Local Government Board will show the members that a little real charity in the consideration of such faults and sins is desirable. We express no opinion on the action, or rather want of action, of the Guardians, but we merely wish to point out that such action—or, again, such want of action—has possibly been discussed at too great a length by the lay Press, which is as incapable of appreciating the difficulties which an infirmary officer may encounter in his treatment of a case, as the Guardians have shown themselves to be of realising the helplessness of a taking-in medical officer whose beds are full and who is forced to send a patient with "query mastoid, query enlarged glands" to the infirmary.

### AN INTERESTING MEETING.

The programme of the American Hospital Association, which meets for its twelfth annual congress on Tuesday, September 20 next, at St. Louis, Missouri, presents many interesting features, and should afford important data for all those who are concerned with hospital work. Two special committees, for instance, will present reports to this congress, which are of more than ordinary interest in view of the fact that their investigations deal with some important sociological problems which largely affect institutional politics. One committee, appointed last year, has inquired into the feasibility of establishing a permanent bureau for the Association, which will be a central station for hospital work in the States. The members of the committee are Dr. S. Goldwater, the well-known chief of the Mount Sinai Hospital at New York; Dr. Hurd, of the Johns Hopkins; and Dr. Truesdale, of the Fall River Hospital. We understand that the report will support the suggestion that such a central institutional bureau is not only desirable but practicable, and the arguments in support of the innovation will be listened to attentively by all those who are in favour of the objects which the Association represents. The second committee has investigated the education and training of nurse-assistants for the care of people of limited means and patients suffering from chronic diseases in the patients' own homes. This inquiry has been made in order to arrive at some conclusion with regard to the position of what we in this country would call district nursing in relation to the large hospitals, and especially their out-patient departments, and the findings of the committee will be of interest not only to hospital workers, and those specially concerned with institutional work, but to physicians and surgeons in general. Dr. Young, of the Presbyterian Hospital, which has a very large nursing school; Miss Riddle, matron of the Johns Hopkins Hospital; and Dr. Washburn, of the Boston General Hospital, have worked on this committee. We hope to give our readers a summary of the two reports when they are presented, and of the discussion that will inevitably follow their publication. Both questions are exciting a lively interest in America, and, although the conditions are not exactly similar on this side, the discussion will doubtless afford valuable information to workers in Great Britain.

### SOME PAPERS FOR HOSPITAL WORKERS.

The addresses already notified and arranged for present many interesting points for discussion. Dr. Hurd will deal with "The Relationship of Trustees to Superintendents," Dr. Fisher with "Private Rooms in General Hospitals," Dr. Washburn with "The Training of Hospital Superintendents and Heads of Departments," Mr. Mucklow, the Director of St. Luke's Hospital at Jacksonville, with "The Preparation of Detailed Reports for Smaller Hospitals," Miss Jaquith, Superintendent of the Memorial Hospital at Worcester, Mass., with the important question of "The Methods of Raising Funds for a General Hospital," and Dr. Beard, who is Secretary of the Minneapolis University Hospital, with "The Whole Question of the Nursing Education in the States." While these are more specialised papers, there are others whose scope and importance are much more general. A paper of decided usefulness is that which will be read by Mr. Burrit, the energetic Secretary of the State Charities Aid Association of New York City, on "Co-operation *versus* Individualism in the Care of the Sick"—a subject that is as much to the fore in America as it promises to become here. Mr. Sutton, the editor of the *International Hospital Record*, will discuss "Hospitals as Commercial Factors," and as Mr. Sutton is well known as an expert who takes a broad, liberal view of hospital work and who has familiarised himself with the various institutional systems available for comparative study, his paper ought to be suggestive and valuable. Dr. Wayne Smith's paper on "Hospital Construction in St. Louis" will appeal more especially to those who are acquainted with the interesting novelties of hospital building in that city, and will be of service for purposes of comparison. In addition there are a number of interesting points, ranged under the general heading of "Question Box," which will afford interesting discussion.

### THE DRUG MARKET.

The market has been exceedingly dull during the week, and price changes have been few. Until the end of September or the beginning of October no general improvement is expected. Opium and its alkaloids continue to be watched with interest, but here in London there is at present no eagerness to make large purchases of opium. There has been a good demand for cream of tartar and tartaric acid at slightly advanced prices. New henbane leaves are tending slightly cheaper.

